# Resilience promotes self-esteem in children and adolescents with hearing impairment: the mediating role of positive coping strategy

**DOI:** 10.3389/fpsyg.2024.1341215

**Published:** 2024-12-20

**Authors:** Ling Qi, Hongling Zhang, Rong Nie, Yukai Du

**Affiliations:** ^1^College of Medicine and Health Science, Wuhan Polytechnic University, Wuhan, China; ^2^Department of Maternal and Child Health, School of Public Health, Tongji Medical College, Huazhong University of Science and Technology, Wuhan, Hubei, China

**Keywords:** children and adolescents, hearing impairment, resilience, self-esteem, positive coping

## Abstract

**Background:**

The level of self-esteem in adolescents appears to be contingent upon their satisfaction across various domains of life, exerting a notable influence on their mental wellbeing. The purposes of this study were to further validate the influence of resilience and positive coping strategy on their self-esteem and to explore the mediating effect of positive coping strategy in the relationship between resilience and self-esteem.

**Methods:**

A total of 657 children and adolescents with a hearing impairment from 14 deaf/special schools in Hubei province completed measures of self-esteem, resilience, and positive coping.

**Results:**

(1) Self-esteem is positively correlated with resilience and positive coping, (2) resilience is a significant predictor of self-esteem, and (3) the association between resilience and self-esteem was partly mediated by a positive coping strategy.

**Conclusion:**

This study indicated the partly mediating effects of positive coping strategy on the association between resilience and self-esteem among Chinese children and adolescents with hearing impairment. These results also highlighted that intervention to promote resilience and coping strategies might be helpful to improve their self-esteem.

## 1 Introduction

The lives of individuals with hearing impairment (HI) are known to be affected by their hearing loss. For children and adolescents with HI, these impacts can be particularly challenging, including worse outcomes in speech, language, education, social functioning, cognitive abilities, and quality of life (Lieu et al., 2020). They frequently face obstacles in communication and limited opportunities for interaction, making them more susceptible to issues in social and emotional functioning. Additionally, co-occurring cognitive and physical impairments may further exacerbate mental health challenges in this population (Stevenson et al., 2015). However, this is not a consistent finding across studies. According to the result of a narrative review, the rates of emotional and behavioral problems in children with HI estimates varied between 0 and 77% (Van Gent et al., 2007). Hence, it is imperative to comprehend the factors and mechanisms that contribute to these variations, as this knowledge will assist practitioners in designing effective interventions for children and adolescents with HI.

The development of the self has been considered an indicator of psychosocial adjustment in children across age, sex, and cultures (Martínez et al., 2007; García et al., 2018; Chen et al., 2020; Fuentes et al., 2020). A core objective during early experiences is for children to develop their self-concept (Rosenberg, 1965; Garcia et al., 2018; Fuentes et al., 2022). In the study of the self, self-esteem as a unidimensional and global component, and self-concept as a multidimensional and specific component have been differentiated (Rosenberg, 1965; Chen et al., 2020). Cross-cultural research has examined the impact of culture on self-concept. As such, collectivism and individualism are aspects that influence the way self-concept is perceived (Singelis et al., 1995; Chen et al., 2004; Markus and Kitayama, 1991). Recognizing these cultural influences is essential, as they can impact the emotional and social wellbeing of children and adolescents, particularly those with hearing impairment. Understanding the relationship between self, culture, and psychosocial adjustment can help practitioners develop more effective interventions that promote healthy development. However, there is relatively limited research in China.

Between the ages of 10–19 years, the phase of adolescence is a critical period of physical and psychological development. During adolescence, self-esteem can fluctuate significantly, and there is a higher chance of experiencing low self-esteem, especially after the age of 13 years (Murray et al., 2017). This risk is further exacerbated if they are HI (Guillon et al., 2003; Polat, 2003; Warner-Czyz et al., 2015). However, the relationship between HI and self-esteem is still unclear.

Self-esteem used to be defined as a personal opinion of oneself and how to value oneself (positive or negative; Wagner et al., 2018). As a multidimensional concept, it serves as a fundamental aspect of quality of life that influences an individual's thoughts, emotions, and behaviors (Sahli and Belgin, 2006). Positive self-esteem can be considered a crucial aspect of mental health (Veselska et al., 2009) and is widely used as an indicator of an individual's overall psychosocial functioning and psychological wellbeing (Baumeister et al., 2003; Trzesniewski et al., 2006). Research with adolescents has found that negative self-esteem is closely related to a wide range of physical, mental, academic, and emotional problems. Given that the important role of self-esteem in the mental and developmental of youth has been well-documented (McClure et al., 2010), comprehending the elements that safeguard and enhance self-esteem becomes imperative. Following on the previous studies, the current study primarily aims to examine the relationships among the resilience, social support and self-esteem, and the mediators in these relationships. Theoretical frameworks and empirical studies (Manne et al., 2015; Gloria and Steinhardt, 2016) support self-esteem as an essential indicator of psychological wellbeing and adaptation, especially in adolescence. This study will, therefore, contribute to the development of prevention and intervention programs aimed at improving self-esteem of adolescent with HI.

Additionally, while the existing research has confirmed the correlation between resilience and self-esteem, it remains unclear what factors drive this connection and which mechanisms are responsible for it. Furthermore, it is also uncertain what potential mediating variables can account for the association between resilience and self-esteem (Kong et al., 2012). This study draws on multiple theoretical perspectives to explain resilience and its connection to self-esteem. According to the broaden and build theory of positive emotion (Gloria and Steinhardt, 2016), resilient individuals identify or cultivate resources and strengths to flexibly handle stressors. In addition, the ecological systems theory (Bronfenbrenner, 2000) highlights the influence of environmental factors, such as family and school, on resilience development in children and adolescents. The transactional model of stress and coping (Lazarus, 1984) further conceptualizes resilience as a process shaped by an individual's appraisal of stress and their coping responses. Resilient individuals employ specific cognitive, behavioral, and affective management strategies in order to adapt and sustain their resilience while regulating their affective response. Together, these frameworks provide a comprehensive view of resilience, emphasizing both internal and external factors that contribute to the psychological wellbeing and adaptive capacities of children and adolescents with HI. These perspectives suggest that positive coping may play a critical role in the resilience–self-esteem relationship by mediating how individuals respond to stressors.

In summary, three main purposes guide our study. First, this study will describe the level of self-esteem of the children and adolescents with HI. Second, the study will explore the connections among resilience, positive coping, and self-esteem. Third, our objective is to explore the potential intermediary function of positive coping in the impact of resilience on self-esteem. Based on empirical and theoretical evidence (Yu et al., 2014), we hypothesized that self-esteem would have a positive correlation with resilience and positive coping. Additionally, resilience and positive coping are both important predictors of self-esteem, as they contribute significantly to enhancing an individual's psychological wellbeing. Furthermore, positive coping mediates the impacts of resilience on self-esteem.

## 2 Materials and methods

### 2.1 Participants and procedures

We used a quantitative approach to collect data in this descriptive cross-sectional study. This study was conducted in Hubei province in China. There are typically three main types of school placement options for HI students in Hubei: (1) Mainstream Schools, where they attend classes alongside students with normal hearing. (2) Special schools, where they coexist with students who have different disabilities, albeit in separate classrooms. (3) Deaf schools, specifically designed for students who have hearing impairments.

All participants in this study are from special schools or schools for the deaf in Hubei province. These students had severely impaired hearing and relied on sign language as their main means of communication. All of the schools are public institutions. Deaf/special schools accept students who have audiogram results from government-recognized hospitals.

To select participants, we employed a two-stage stratified cluster sampling method across eight regions in Hubei province, resulting in the inclusion of 14 deaf/special schools. To ensure that all participants possess sufficient proficiency to comprehend the statements in the questionnaire, it is required that all participants have received a minimum of 6 years of formal education. Those with cognitive impairments or health complications, as well as those who refused to take part in the survey, were excluded. During the questionnaire administration, both the interviewer and a sign language interpreter were present to facilitate communication.

### 2.2 Ethical considerations

This study was approved by the ethical committee of Tongji Medical College, Huazhong University of Science and Technology. After the acquisition of consent from the relevant authorities at each school, a teacher proficient in sign language conveyed the objectives of the research and other details outlined in the authorized informed consent forms to the students. Moreover, it was clarified that they have the right to withdraw at any given moment. A show of hands was used to confirm that the students understood the study and indicated their willingness to participate. Subsequently enrolled were only the students who willingly participated in the study.

### 2.3 Measure

#### 2.3.1 Socio-demographic and clinical characteristics

Data about demographic and injury characteristics were collected using a self-administered questionnaire, including age, gender, ethnicity, education level, age of hearing impairment identification, severity of injury, history of rehabilitation, parental education, parenting style, and daily parent-child interaction. Peer relationships and academic performance were treated as categorical variables, classified into four levels: excellent, good, average, and poor.

#### 2.3.2 Self-esteem

Self-esteem was measured using the Chinese version of the Rosenberg self-esteem scale. This self-report scale includes 10 statements, with respondents rating their agreement or disagreement on a 4-point Likert scale (1 = strongly disagree to 4 = strongly agree). Items 1, 2, 4, 6, and 7 were the statements about positive self-evaluation (e.g., I take a positive attitude toward myself). Items 3, 5, 8, 9, and 10 were the statements about negative self-evaluation (e.g., I feel I do not have much to be proud of). In this scale, higher scores (after converting reverse items) indicate higher levels of self-esteem. In the current study, the coefficient alpha was 0.70.

#### 2.3.3 Resilience

Participant resilience was assessed using the Resilience Scale for Chinese Adolescents (RSCA; Hu and Gan, 2008). This scale contains 27 items, with each item rated on a five-point Likert scale (1: strongly disagree to 5: strongly agree). Respondents indicated the extent to which they agreed with statements that evaluated their personal resilience or ability to recover from stress. The RSCA is divided into five dimensions: goal setting, emotional control, positive cognition, interpersonal assistance, and family support. The mean scores are used to measure resilience, with higher scores indicating higher resilience levels. This scale demonstrated good to excellent internal reliability as reported by previous research (Gloria and Steinhardt, 2016), and the present study also found the scale to be reliable with a coefficient alpha of 0.86.

#### 2.3.4 Coping behaviors

The Simplified Coping Style Questionnaire (SCSQ; Xie, 1998) was used to evaluate the coping behaviors of participants in this study. The positive coping (12 items, e.g., I try to think of different ways to solve a specific problem), rated on a four-point Likert scale (0–3), with higher scores representing greater use of the particular strategy (Yu et al., 2014). The coefficient alpha for the SCSQ-P was 0.80 in this study.

### 2.4 Method of data analysis

Descriptive statistics were computed for all variables. Continuous data were summarized using the mean and standard deviation, while categorical data were summarized using percentages. The correlations of self-esteem with socio-demographic and clinical variables were examined using univariate analyses. One-way ANOVA was used to analyze differences in self-esteem scores across various levels of peer relationships and academic performance. To examine the connections between self-esteem, resilience, and coping styles, Pearson correlation analysis was conducted. Additionally, hierarchical linear regression analysis was used to assess the impact of resilience and positive coping on self-esteem.

To examine the mediating role of positive coping in the relationship between resilience and self-esteem, we conducted a mediation analysis following the guidelines of Baron and Kenny (1986). Sobel's *z*-test was used to assess the significance of the indirect effect, which confirms mediation when the *z*-value is significant. Partial mediation was concluded if the relationship between resilience and self-esteem remained significant but was reduced when positive coping was included as a mediator.

## 3 Results

### 3.1 Basic information

Among the 657 participants, 54.3% were male and 44.8% were female, the remaining 0.9% had missing data. The average age of the students involved in the study was 17.26 ± 3.51 years. Out of the total students, 32.4% experienced congenital permanent hearing impairment, while 41.9% had acquired permanent hearing impairment. Additionally, 25.7% of students reported uncertainty about the age when their hearing impairment began. According to the classification and grading criteria of disability released in 2001 by the Standardization Administration of China (GB/T 26341-2010; Qi et al., 2020). Out of the 657 participants involved, the combination of severe and profound hearing impairment made up 80.2% of the total, while the combination of mild and moderate degrees accounted for 7.3% (excluding 82 missing cases). A slight majority of participants (55.6%) received hearing rehabilitation, while 37.7% of participants did not receive any hearing rehabilitation. The majority of students' parents' educational levels were high school.

As for participants' subject perception, 69.1% (including those who responded “very good” and “good”) of participants reported that they get along well with their peers, and 2.9% of students reported they could not. Considering academic performance, 48.9% of participants reported “general,” followed by 34.1% reporting “good,” 12.3% “excellent” and 4.0% “bad” in academic performance (missing 5).

### 3.2 Univariate analyses

The correlations of self-esteem with socio-demographic and clinical variables are shown in Table 1. Self-esteem represents a key indicator of successful coping with the developmental tasks of adolescence. The mean and SD for self-esteem were 26.1 and 4.1, respectively. The mean self-esteem total score indicated these HI participants reported a slightly higher self-esteem level than the median level (possible range 10–40 with median 25). The one-way ANOVA showed that the mean self-esteem scores varied significantly in the different peer relationships and academic performance (both *P* < 0.05). Furthermore, a two-sample *t*-test showed the mean self-esteem score for participants with congenital HI was higher than that reported by participants with acquired HI (*t* = 1.93, *P* = 0.05).

**Table 1:** Results of self-esteem scores according to the independent variables examined.

	***N***	**Self-esteem (mean + SD)**	***t/F***	***P***
**Sex**				
Male	355	26.18 ± 4.13	0.06	0.95
Female	293	26.16 ± 3.87		
**Grade**				
Junior Middle school	282	25.92 ± 3.95	−1.56	0.12
Senior Middle school	343	26.43 ± 4.17		
**Degree of hearing loss**				
Profound (>90 dB HL)	404	25.90 ± 4.08	1.26	0.29
Severe (81–90 dB HL)	123	26.69 ± 4.35		
Moderate (61–80 dB HL)	36	26.33 ± 3.98		
Mild (41–60 dB HL)	12	25.58 ± 3.42		
**Age of identification of hearing impairment**				
Congenital	213	26.64 ± 4.24	1.93	0.05
Acquired	275	25.34 ± 0.44		
**Hearing rehabilitation received**				
Yes	365	26.40 ± 4.15	1.47	0.14
No	248	25.91 ± 3.94		
**Maternal educational level**				
≤ Elementary	219	26.21 + 3.93	0.85	0.43
High school	380	26.19 + 4.18		
≥College	58	25.47 + 3.93		
**Paternal educational level**				
≤ Elementary	115	26.12 ± 3.63	0.21	0.81
High school	472	26.18 ± 4.26		
≥College	70	25.84 ± 3.48		
**Peer relationship**				
Very good	165	26.95 ± 3.65	3.60	0.01
Good	289	26.09 ± 4.30		
General	177	25.37 ± 4.08		
Poor	19	26.42 ± 3.10		
**Academic performance**				
Excellent	81	27.27 ± 3.54	3.21	0.01
Good	224	26.42 ± 4.07		
Average	321	25.69 ± 3.58		
Bad	26	26.14 ± 4.08		

Correlation analyses were carried out to evaluate the main variables (resilience, positive coping) and self-esteem. The results are shown in Table 2. According to the Pearson correlation analysis, self-esteem showed a significant positive correlation with resilience (RSCA, *r* = 0.27, *P* < 0.01) and specifically with the goal setting subscale (*r* = 0.20, *P* < 0.01). Other subscales, such as positive cognition and family support, demonstrated positive correlations with self-esteem but did not reach statistical significance. Furthermore, a significant positive correlation was found between resilience and positive coping (all *P* < 0.01). We then performed a hierarchical linear regression analysis to test the contributions of resilience and positive coping on self-esteem. In the initial stage, three variables were included (age of identification of hearing impairment, peer relationship, and academic performance). Resilience was entered in Step 2. As detailed in Table 3, greater resilience predicted a higher self-esteem score. A study reported that the effect of positive coping on self-esteem was significant when it was introduced in the third step (B = 0.082, S.E. = 0.030, *P* < 0.01). Resilience and positive coping could account for 8.3% of the overall variation in self-esteem.

**Table 2:** Mean, standard deviations, and correlations among the study's main variables.

	**Mean ±SD**	**RSCA**	**Goal setting**	**Emotional control**	**Positive cognition**	**Interpersonal assistance**	**Family support**	**SCSQ-P**
Self-esteem	26.13 ± 4.08	0.27^**^	0.20^**^	0.20	0.21	0.19	0.20	0.26
RSCA	3.23 ± 0.54	1	0.75	0.68	0.73	0.80	0.73	0.56
Goal setting	3.02 ± 0.79		1	0.30	0.63	0.44	0.37	0.55
Emotional control	3.03 ± 0.74			1.00	0.30	0.55	0.39	0.34
Positive cognition	3.37 ± 0.77				1.00	0.45	0.73	0.51
Interpersonal assistance	3.05 ± 0.66					1.00	0.50	0.39
Family support	3.23 ± 0.69						1.00	0.29

^**^P < 0.01.

RSCA, Resilience Scale of Chinese Adolescent; SCSQ-P, Positive coping subscale of the Simplified Coping Style Questionnaire.

**Table 3:** Hierarchical regression analysis: prediction of self-esteem.

	**Step 1**			**Step 2**			**Step 3**		
	**B**	**SE**	***t***	**B**	**SE**	***t***	**B**	**SE**	***t***
Age onset of hearing loss	0.008	0.004	2.173^*^	0.009	0.004	2.519^*^	0.010	0.004	2.708^**^
Peer relationship	0.430	0.207	2.074^*^	0.321	0.200	1.602	0.280	0.200	1.401
Academic performance	0.560	0.218	2.558^*^	0.607	0.210	2.890^**^	0.548	0.210	2.614^**^
RSCA				2.031	0.284	7.159^***^	1.464	0.340	4.302^***^
SCSQ-P							0.082	0.030	0.003^**^
*R* ^2^	0.180			0.321			0.339		
Δ*R*^2^				0.071			0.012		
*F*	7.26^***^			18.68^***^			16.90^***^		

RSCA, Resilience Scale of Chinese Adolescent; SCSQ-P, Positive coping subscale of the Simplified Coping Style Questionnaire.

^*^P < 0.05; ^**^P < 0.01; ^***^P < 0.001.

### 3.3 The mediating role of positive coping

In order to examine whether positive coping is a mediator of the relationship between resilience and self-esteem (Yu et al., 2014), mediation tests were performed according to the procedure set by Baron and Kenny's mediation steps and Sobel's *Z*-test (Baron and Kenny, 1986). First, we calculated the unstandardized coefficient and standard error (S.E.) for the pathway from the predictor to the mediator by regressing positive coping onto resilience, while adjusting for age at identification of hearing impairment, peer relationships, and academic performance (B = 6.885, S.E. = 0.040, *P* < 0.01). Then, we calculated the unstandardized coefficient and S.E. for the pathway from the mediator to the dependent variable (B = 0.082, S.E. = 0.030, *P* < 0.01). Sobel's *Z*-test was applied to test the indirect effect, yielding a significant *z*-value, which confirms the mediation effect of positive coping. This partial mediation is further supported by the reduction in the direct effect of resilience on self-esteem (from B = 2.031, *P* < 0.001 to B = 1.464, *P* < 0.01) when positive coping was included in the model. These results indicated that resilience is indirectly related to self-esteem through positive coping (Effect M = 27.82%). The analysis confirmed that positive coping partially mediates the relationship between resilience and self-esteem, as the direct effect of resilience on self-esteem remained significant but was reduced when positive coping was included as a mediator.

## 4 Discussion

To our knowledge, this is one of the few studies investigating self-esteem in adolescents with HI in China. The first objective of the present study was to investigate self-esteem in adolescents with HI. The results of the present study suggested that the self-esteem levels of adolescents with HI were lower than those in previous studies based on children and adolescents with HI (Mean = 22.52, SD = 5.26 on a scale ranging from 0 to 30; Warner-Czyz et al., 2015), young people with epilepsy (Mean = 28.78, SD = 6.04; Chew et al., 2017), adolescents with specific language impairment (Mean = 30.55, SD = 4.95; Wadman et al., 2008). Furthermore, children and adolescents with HI (M = 26.1, SD = 4.1) rated global self-esteem as lower than peers in the general population (18,612 junior high and high school students), as published by Dukes and Martinez (1994; M = 30.75, SD = 5.06). The findings align with Polat (2003) suggesting that teenagers with HI have greater obstacles in terms of their self-worth due to frequently confronting various hurdles, including delays in speech and language, difficulties in communication, and limited or no exposure to the predominantly auditory environment. Furthermore, the situation becomes more critical for older adolescents, as this age group is transitioning from school to the workplace. The mean age of adolescents with HI (Mean = 17.26 years) in our study was older than in previous similar studies, which may explain the inconsistent results. Considering the other findings of our study, such as the differences in social interactions and academic performances among adolescents with HI, another explanation for the lower self-esteem in adolescents with HI can be given. Particularly type of school and self-esteem level appeared to be very relevant. Adolescents attending special schools showed lower self-esteem than those in mainstream schools (Van Gurp, 2001; Lesar and Vitulic, 2014; Theunissen et al., 2014). However, no consensus has been reached regarding the effect of educational setting on the self-esteem of children with HI (Keilmann et al., 2007; Theunissen et al., 2014). In line with previous studies (Erol and Orth, 2011; Lesar and Vitulic, 2014), the level of hearing loss did not appear to affect the self-esteem of adolescents with HI in our study. Adolescents with hearing impairment do not possess differences in self-esteem solely based on the degree of their hearing loss. Furthermore, there were no difference between gender (girl vs. boy) and grade (junior vs. senior middle school). According to Crowe's interpretation (Crowe, 2003), this outcome may be attributed to the notion that both girls and boys from HI backgrounds are brought up in a nurturing and supportive setting, leading them to assess themselves on an equal basis. There are similar levels of self-esteem among adolescents in different grades, suggesting that development of self-esteem does not significantly change during adolescence (Lesar and Vitulic, 2014).

Our second objective was to evaluate correlations among resilience, positive coping, and self-esteem. Pearson's correlation analysis showed that positive significant low/moderate correlations of self-esteem with resilience (included total and five dimensions scores) and positive coping had been observed. These findings indicate that the participants who reported high scores on self-esteem showed higher resilience as well as positive coping, which clearly supports the hypothesis that resilience and positive coping are related to self-esteem. Resilience and positive coping, as indicated by previous research (Benetti and Kambouropoulos, 2006), are reliable indicators of self-esteem. Furthermore, hierarchical regression analysis showed resilience and positive coping were significant predictors of increased self-esteem when controlling the age of identification of HI, peer relationships, and academic performance. In our study, the contribution of resilience to self-esteem was found to be above the effect of positive coping. It concurs with previous research (Feggi et al., 2016; Li et al., 2020), emphasizing the significance of resilience in promoting self-esteem. A further finding from the hierarchical regression analysis showed that age, along with HI and academic performance (based on self-report), were significant predictors of increased self-esteem in model 3 (see Table 3). The age at which hearing impairment (HI) onset was positively, though weakly, related to the self-esteem of adolescents with HI. That is to say, a later onset of hearing loss was associated with higher self-esteem. The present study's findings are consistent with previous findings, but there appears to be inconsistency in the literature on the impact of age onset of hearing loss on the self-esteem of HI individuals (Polat, 2003; Dammeyer et al., 2019). Academic performance was found to be another powerful predictor of the study. HI students who performed better in academic performance were also known to have better self-esteem. However, like all the other variables discussed so far, the effect of academic performance on self-esteem has been controversial. There may be complexities of factors influencing the relationship between academic performance and self-esteem, which need further research.

The third objective of our study was to investigate the effect of positive coping on the relationship between resilience and self-esteem of adolescents with HI. The findings from mediation analysis in the current study revealed that resilience directly affects self-esteem; and is partly mediated via the simple mediator of positive coping. These results indicated that resilient individuals were more likely to respond to stress by positive coping strategies. This finding supports previous research that identifies positive coping as a critical factor in psychological wellbeing (Dammeyer et al., 2019; Dong et al., 2024). Indeed, previous studies have indicated there may be three strategies (namely, positive emotional expression, positive reappraisal, and cultivation of a sense of peace and meaning), which may be most successful in maintaining psychological adjustment to other challenging situations. For example, a previous study found that resilience is associated with positive reappraisal, where resilient individuals are more likely to use specific cognitive strategies to assimilate difficult life experiences (Manne et al., 2015). In turn, positive reappraisal leads to better self-esteem.

Overall, the findings from the current study build on previous research on the self-esteem of adolescents with HI by providing preliminary evidence for the mediating effect of positive coping in the relationship between resilience and self-esteem. These findings highlight the beneficial effect of resilience and positive coping on self-esteem. Thus, intervention experts and therapists can mainly focus on techniques that enhance the resilience of adolescents with HI and positive coping strategies to improve their self-esteem and mitigate the negative effect of HI on self-esteem.

There are some limitations to the study that should be taken into consideration. First, the study was cross-sectional, meaning the direction of causation cannot be determined. Longitudinal studies are needed to shed light on the causal relationships between resilience, positive coping, and self-esteem. Second, participants in our study were recruited from Hubei province and were all affiliated with deaf/special schools. As a result, caution is needed when generalizing the findings to HI youth from other areas or those not attending deaf/special schools. For example, HI youth who are home-based may have different levels of resilience, positive coping, and self-esteem. Further research is needed with more diverse samples to strengthen the generalization of findings from our study.

## Data Availability

The raw data supporting the conclusions of this article will be made available by the authors, without undue reservation.
